# Supplementary material for: Fear of Covid 19 during the third wave of infection in Norwegian patients with type 1 diabetes
Source: PLoS One. 2022 Jul 28;17(7):e0272133. doi: 10.1371/journal.pone.0272133 (PMC9333287; doi:10.1371/journal.pone.0272133)
Supplement: Attachment — Submitted filename: Point to point rebuttal letter Fear of covid 19 .docx [file pone.0272133.s001.docx]

Point-to-point rebuttal letter

Thank you for the constructive comments on our manuscript PONE-D-22-07616

“Fear of Covid 19 during the third wave of infection in Norwegian patients with type 1 diabetes”

We have now carefully revised the paper according to the comments by the reviewer as detailed below. Changes in the manuscript are made in red. We hope you will find the revised version acceptable for publication.

Editor Comments: The paper should be checked by a professional speaker of English before complete acceptance.

The paper has now been checked by last author of the manuscript, John Cooper, who is a native Englishman.

Journal Requirements:

When submitting your revision, we need you to address these additional requirements.

1. Please ensure that your manuscript meets PLOS ONE's style requirements, including those for file naming. The PLOS ONE style templates can be found at <https://journals.plos.org/plosone/s/file?id=wjVg/PLOSOne_formatting_sample_main_body.pdf> and 
<https://journals.plos.org/plosone/s/file?id=ba62/PLOSOne_formatting_sample_title_authors_affiliations.pdf>

The manuscript is now formatted according to PLOS Ones style requirements.

2. Please provide additional details regarding participant consent. In the ethics statement in the Methods and online submission information, please ensure that you have specified (1) whether consent was informed and (2) what type you obtained (for instance, written or verbal, and if verbal, how it was documented and witnessed). If your study included minors, state whether you obtained consent from parents or guardians. If the need for consent was waived by the ethics committee, please include this information.

If you are reporting a retrospective study of medical records or archived samples, please ensure that you have discussed whether all data were fully anonymized before you accessed them and/or whether the IRB or ethics committee waived the requirement for informed consent. If patients provided informed written consent to have data from their medical records used in research, please include this information.

All the participants have given written informed consent. This information is now included in the method section, page 7 line 142.

3. In your Data Availability statement, you have not specified where the minimal data set underlying the results described in your manuscript can be found. PLOS defines a study's minimal data set as the underlying data used to reach the conclusions drawn in the manuscript and any additional data required to replicate the reported study findings in their entirety. All PLOS journals require that the minimal data set be made fully available. For more information about our data policy, please see <http://journals.plos.org/plosone/s/data-availability>.

"Upon re-submitting your revised manuscript, please upload your study’s minimal underlying data set as either Supporting Information files or to a stable, public repository and include the relevant URLs, DOIs, or accession numbers within your revised cover letter. For a list of acceptable repositories, please see <http://journals.plos.org/plosone/s/data-availability#loc-recommended-repositories>. Any potentially identifying patient information must be fully anonymized.

Important: If there are ethical or legal restrictions to sharing your data publicly, please explain these restrictions in detail. Please see our guidelines for more information on what we consider unacceptable restrictions to publicly sharing data: <http://journals.plos.org/plosone/s/data-availability#loc-unacceptable-data-access-restrictions>. Note that it is not acceptable for the authors to be the sole named individuals responsible for ensuring data access.

We will update your Data Availability statement to reflect the information you provide in your cover letter.

Sharing of individual participant data with third parties was not specifically included in the informed consent of the study, and unrestricted distribution of such data may pose a potential threat of revealing participants’ identities. To minimise this risk, researchers who wish to inquire about access to individual participant data that underlie the results reported in this article can submit a request to the The Norwegian Adult Diabetes registry (noklus @noklus.no). To gain access, researchers will need to sign a data access agreement and obtain the approval of the local ethics committee.

4. Please include a copy of Table 2 which you refer to in your text on page 7.

We apologize for the mistake, as Table 2 was identical with Supplementary Table S1. We have now chosen to include Supplementary Table S2 in the main manuscript, as Table 3. We no longer need a supplementary file. The alterations in the numbering of tables are now marked in red throughout the manuscript.

5. Please include captions for your Supporting Information files at the end of your manuscript, and update any in-text citations to match accordingly. Please see our Supporting Information guidelines for more information: <http://journals.plos.org/plosone/s/supporting-information>

Supporting/supplementary file is no longer included in the manuscript.

Reviewers' comments:

Reviewer's Responses to Questions

**Comments to the Author**

1. Is the manuscript technically sound, and do the data support the conclusions?

The manuscript must describe a technically sound piece of scientific research with data that supports the conclusions. Experiments must have been conducted rigorously, with appropriate controls, replication, and sample sizes. The conclusions must be drawn appropriately based on the data presented.

Reviewer #1: Partly

2. Has the statistical analysis been performed appropriately and rigorously?

Reviewer #1: No

3. Have the authors made all data underlying the findings in their manuscript fully available?

The [PLOS Data policy](http://www.plosone.org/static/policies.action#sharing) requires authors to make all data underlying the findings described in their manuscript fully available without restriction, with rare exception (please refer to the Data Availability Statement in the manuscript PDF file). The data should be provided as part of the manuscript or its supporting information, or deposited to a public repository. For example, in addition to summary statistics, the data points behind means, medians and variance measures should be available. If there are restrictions on publicly sharing data—e.g. participant privacy or use of data from a third party—those must be specified.

Reviewer #1: No

4. Is the manuscript presented in an intelligible fashion and written in standard English?

PLOS ONE does not copyedit accepted manuscripts, so the language in submitted articles must be clear, correct, and unambiguous. Any typographical or grammatical errors should be corrected at revision, so please note any specific errors here.

Reviewer #1: No

5. Review Comments to the Author

Reviewer #1: 1. Grammatical, formatting and spacing mistakes should be corrected in the manuscript. Font color should be black.

We have reviewed the manuscript carefully, and hope that all grammatical, formatting and spacing mistakes are corrected. Font color is black.

2. Introduction should include background of COVID-19 and type 1 diabetes especially prevalence, symptoms of COVID-19, its main variants and the role of immunity in prevention of this viral disease. Following articles can be used as references:
(Endocr Metab Immune Disord Drug Targets. 2022; <https://doi.org/10.2174/1871530322666220110113028>).
(J Med Virol. 2021; <https://doi.org/10.1002/jmv.27256>).

Thank you for this valuable suggestion of improvement, we have now included this in the introduction page 3, line 47-53 and page 4 line 73-74.

3. Reference format should be modified according to journal.

We have now modified the reference to style Vancouver as required.

4. Most of the participants were male (54.1%) in your study but fear factor is more in female then man. justify this statement.

You are correct, there are more men than women with type 1 diabetes in Norway, and our study population is therefore representative for the actual patient population. In addition, we have a high number of study participants, and the statistical power is high- so the fact that more men than women are participating is not affecting the results.

5. Result is too long, try to trim it by removing extra explanation.

Thank you- we have now trimmed the results section from the original 854 words to 734 words.

6. Be specific with your topic and shorten your discussion up to 2 pages. References are also missing in most of the sentences in discussion.

We agree, the discussion was too long. We have shortened it from 1862 words to 1445 words, which corresponds to 4 pages instead of 6 (discussion without conclusion).

7. What are the limitations of your study?

We agree that the limitations of the study should have been presented more explicitly. We have addressed this problem with a revised section on page 14, line 315-322:

The results of the study should be interpreted in light of the prevalence of COVID 19 in the country at the time of the data collection. In Norway, the first cases of COVID 19 were confirmed in February 2020. The peak of the outbreak was during March and April 2020. The third wave of infection in Norway started in the middle of March 2021, and as of the 1st of May 2021 (when the fear questionnaire was sent out), the total number of confirmed Covid 19 cases was 113899 and there had been 766 deaths from Covid 19 (31). The relatively low level of viral transmission in the society when the questionnaire was distributed, and the digital collection of data could have biased our results, and is an important limitation of the study.

The large number of participants could make the study “over powered”, finding statistic significances that are rather weak and not clinically relevant.

8. What is the future perspective of your study?

Regarding future perspective of the study, we have presented the findings of this study at a national meeting for both doctors and nurses- to encourage vigilance in this area at diabetic outpatient clinics and, if necessary, to provide psychological help to those with a high level of fear of Covid 19.

Furthermore we have distributed a much larger questionnaire to all diabetes patients (both DM1 and DM2) in Norway registered in the national register for adults to assess how these patients have coped with the pandemic overall. The results are to be processed.

In addition, we think that the methodological principle with digital collection of data directly from the patient is a novel and exciting way to enrich clinical studies in the future. (Included in the conclusion of the manuscript)
