# Supplementary material for: Fear of Covid 19 during the third wave of infection in Norwegian patients with type 1 diabetes
Source: PLoS One. 2022 Jul 28;17(7):e0272133. doi: 10.1371/journal.pone.0272133 (PMC9333287; doi:10.1371/journal.pone.0272133)
Supplement: Attachment — Submitted filename: Ueland rebuttal rev 2 Fear of covid 19 08.06.2022 .docx [file pone.0272133.s002.docx]

Point-to-point rebuttal letter

Thank you for the constructive comments on our manuscript PONE-D-22-07616

“Fear of Covid 19 during the third wave of infection in Norwegian patients with type 1 diabetes”

We have addressed the comments of the reviewers in the revised paper. Changes in the manuscript are highlighted in red. The manuscript has been checked for grammar and syntax  by native English speaker. We hope you will find the revised version acceptable for publication.

Reviewer #1:

1. Summarize the whole manuscript comprehensively in the abstract (limit the words up to 250).

We think the abstract comprehensively covers the whole manuscript, and it consists of 240 words.

2. Figure 3 is not clear, re-draw this figure and add caption of graphs as well.

Figure 3 is now re-drawn, and section 3A, 3B and 3C are submitted as individual figures to secure better resolution. All graphs have captions. The figure legend is also updated/improved.

3. As you mentioned that you have cite the suggested paper on page 4 line 73-74, but in your manuscript suggested paper is cited on line 72 with reference # 8 rather than reference # 9. It means the sequence of references is incorrect.?

The sequences of references are correct, however the page and line numbers were incorrect. We have rectified the error.

Suggested reference (Endocr Metab Immune Disord Drug Targets. 2022; [https://doi.org/10.2174/1871530322666220110113028) is reference 1](https://doi.org/10.2174/1871530322666220110113028)%20is%20reference%201), page 3 line 52.

And suggested reference (J Med Virol. 2021; <https://doi.org/10.1002/jmv.27256>) is reference 8 at page 4 line 72.

4. Font color should be black in introduction. Re-check your font setting and correct it.

Font color is black in the introduction.

5. Paragraph setting of whole manuscript is required as well. Paragraph setting should be justified.

We have reassessed the paragraph setting of whole manuscript, and justified it.

6. Try to trim the discussion furthermore (maximum 3 pages).

We have shortened the discussion substantially, and it is now just over 3 pages.

7. Conclude your findings under a separate heading.

We have now established a separate heading for Conclusion (line 298).
