# Supplementary material for: Fear of Covid 19 during the third wave of infection in Norwegian patients with type 1 diabetes
Source: PLoS One. 2022 Jul 28;17(7):e0272133. doi: 10.1371/journal.pone.0272133 (PMC9333287; doi:10.1371/journal.pone.0272133)
Supplement: Attachment — Submitted filename: Ueland rebuttal rev 3 Fear of covid 19 08.06.2022 .docx [file pone.0272133.s003.docx]

Point-to-point rebuttal letter

Thank you for the constructive comments on our manuscript PONE-D-22-07616

“Fear of Covid 19 during the third wave of infection in Norwegian patients with type 1 diabetes”

We have addressed the comments of the reviewers in the revised paper. Changes in the manuscript are highlighted in red. The manuscript has been checked for grammar and syntax  by native English speaker. We hope you will find the revised version acceptable for publication.

Reviewer #1:

1. Summarize the whole manuscript comprehensively in the abstract (limit the words up to 250).

We think the abstract now comprehensively covers the whole manuscript, and it consists of 240 words.

2. Figure 3 is not clear, re-draw this figure and add caption of graphs as well.

Figure 3 is now re-drawn, and section 3A, 3B and 3C are submitted as individual figures to secure better resolution. All graphs have captions. The figure legend is also updated/improved.

If you want the figure to look different, please specify, and we will try to change it further.

3. As you mentioned that you have cite the suggested paper on page 4 line 73-74, but in your manuscript suggested paper is cited on line 72 with reference # 8 rather than reference # 9. It means the sequence of references is incorrect.?

The sequences of references are correct, however the page and line numbers were incorrect. We have rectified the error.

Suggested reference (Endocr Metab Immune Disord Drug Targets. 2022; (https://doi.org/10.2174/1871530322666220110113028) is referred in reference 1, page 3, line 52.

And suggested reference (J Med Virol. 2021; <https://doi.org/10.1002/jmv.27256>) is reference 8 at page 4 line 72.

4. Font color should be black in introduction. Re-check your font setting and correct it.

Font color is changed to black in the introduction, and the whole manuscript.

5. Paragraph setting of whole manuscript is required as well. Paragraph setting should be justified.

**We have reassessed the paragraph setting of whole manuscript.**

**The introduction** is divided into paragraphs without subheadings.

**The method section** is subdivided into paragraphs with the following subheadings: Participants/data collection, Measurements, Fear of Covid 19 scale, Statistical analysis and Ethical considerations.

**The result section** is divided into paragraphs with the following headings:

Study population, Fear score assessed against various demographic and clinical variables, patients that did not answer or answered incomplete.

**The discussion** part is divided into paragraphs without subheadings.

6. Try to trim the discussion furthermore (maximum 3 pages).

We have shortened the discussion substantially from 1632 words to 1047 words, and it is now 3 pages.

7. Conclude your findings under a separate heading.

We have now established a separate heading for Conclusion (line 299).
